# CMIP5 climate projections and RUSLE-based soil erosion assessment in the central part of Iran

**DOI:** 10.1038/s41598-021-86618-z

**Published:** 2021-03-31

**Authors:** Fatemeh Hateffard, Safwan Mohammed, Karam Alsafadi, Glory O. Enaruvbe, Ahmad Heidari, Hazem Ghassan Abdo, Jesús Rodrigo-Comino

**Affiliations:** 1 Department of Landscape Protection and Environmental Geography, Faculty of Science and Technology, University of Debrecen, Debrecen, Hungary; 2 Institute of Land Use, Technology and Regional Development, University of Debrecen, Debrecen, 4032 Hungary; 3 Department of Geography and GIS, Faculty of Arts, Alexandria University, Alexandria, 25435 Egypt; 4 School of Geographical Sciences, Nanjing University of Information Science and Technology, Nanjing, 210044 China; 5 African Regional Institute for Geospatial Information Science and Technology, Obafemi Awolowo University, Ile-Ife, Nigeria; 6 Soil Science Department, University of Tehran, Karaj, Iran; 7 Geography Department, University of Tartous, Tartous, Syria; 8 Geography Department, University of Damascus, Damascus, Syria; 9 Geography Department, University of Tishreen, Lattakia, Syria; 10 Physical Geography, Trier University, 54296 Trier, Germany; 11 Soil Erosion and Degradation Research Group, Department of Geography, University of Valencia, 46010 Valencia, Spain

**Keywords:** Climate sciences, Natural hazards

## Abstract

Soil erosion (SE) and climate change are closely related to environmental challenges that influence human wellbeing. However, the potential impacts of both processes in semi-arid areas are difficult to be predicted because of atmospheric variations and non-sustainable land use management. Thus, models can be employed to estimate the potential effects of different climatic scenarios on environmental and human interactions. In this research, we present a novel study where changes in soil erosion by water in the central part of Iran under current and future climate scenarios are analyzed using the Climate Model Intercomparison Project-5 (CMIP5) under three Representative Concentration Pathway-RCP 2.6, 4.5 and 8.5 scenarios. Results showed that the estimated annual rate of SE in the study area in 2005, 2010, 2015 and 2019 averaged approximately 12.8 t ha^−1^ y^−1^. The rangeland areas registered the highest soil erosion values, especially in RCP2.6 and RCP8.5 for 2070 with overall values of 4.25 t ha^−1^ y^−1^ and 4.1 t ha^−1^ y^−1^, respectively. They were followed by agriculture fields with 1.31 t ha^−1^ y^−1^ and 1.33 t ha^−1^ y^−1^. The lowest results were located in the residential areas with 0.61 t ha^−1^ y^−1^ and 0.63 t ha^−1^ y^−1^ in RCP2.6 and RCP8.5 for 2070, respectively. In contrast, RCP4.5 showed that the total soil erosion could experience a decrease in rangelands by − 0.24 t ha^−1^ y^−1^ (2050), and − 0.18 t ha^−1^ y^−1^ (2070) or a slight increase in the other land uses. We conclude that this study provides new insights for policymakers and stakeholders to develop appropriate strategies to achieve sustainable land resources planning in semi-arid areas that could be affected by future and unforeseen climate change scenarios.

## Introduction

Changes in land uses have consistently been described because of rapid population growth and the expansion of human settlement around the world^1–7^. These changes play important roles in shaping the landscape and altering land resources, sometimes with negative impacts^8^. Numerous scholars have concluded that unregulated land-use changes lead to environmental degradation that poses a major threat to the socioeconomic and ecological sustainability of soil as a vital resource^9–11^. Increasing pressure on land resources because of unsustainable cultivation, overgrazing, deforestation, climate change and drought, urbanization and poor land management practices are worsening land degradation on a global scale^12–15^. Among them, soil erosion (SE) is one of the common forms of land degradation that is related to unsustainable environmental management. Soil erosion is particularly severe in arid and semi-arid regions^15–20^.

SE is a complex process resulting from the impacts of wind, precipitation, human activities and associated runoff processes that are influenced by parent material, soil properties, relief and vegetation cover^21,22^. Although SE may occur naturally, anthropogenic activities such as land-use change, agriculture, livestock grazing or deforestation are known to exacerbate erosion and soil degradation^23–25^. Therefore, SE is considered a natural and human-induced challenge^9,13,22^, that leads to severe adverse socioeconomic and environmental damage in many societies^26,27^. Despite the important implications of SE on sustainable use of soil; however, there is limited information on current and future scenarios. The dearth of this information is linked to the complexity of erosion processes which makes SE estimation expensive, time-consuming and difficult^28,29^. This difficulty has resulted in the development of various models and tools that seek to simplify SE modelling and improve our understanding of the pattern and processes of SE.

The Universal Soil Loss Equation (USLE)^30,31^ model is widely used for estimating SE because it integrates many of the components of the SE process^13,26,29,32,33^. Apart from the anthropogenic factors driving SE, recent studies show that other factors influencing land degradation are climate-related^32,34,35^. On the other hand, the Intergovernmental Panel on Climate Change (IPCC) has launched the four future scenarios for earth greenhouse gases (GHGs) emission, which is known as Representative Concentration Pathways (RCPs) 2.6, 4.5, 6, and 8.5^36^. These scenarios simulate different GHGs emission, the RCP2.6 refer to low GHGs emission, the RCP4.5, and RCP6 express as stabilization scenarios, while RCP8.5 denote high GHGs emission^37^. Studies have been carried out to predict the impact of future climate on soil erosion by using different CMIP5-RCP scenarios (i.e. Tibetan Plateau^38^; Lancang–Mekong River^39^; Minab Dam Watershed^35^; Burkina Faso^40^; mid-Yarlung Tsangpo River region^41^).

SE is a natural geomorphological process (erosion, transport and sedimentation) but after human disturbances can be considered as a land degradation one, which has been a recurring challenge for decades over the world for stakeholders, and especially, in countries such as Iran. Recently, scientists have examined land degradation indicators including desertification^42^, deforestation^43^, salinization^44^, alkalization of soils^45^, overgrazing^46^, intensive land-use changes^47^, and especially, water and wind erosion^48,49^. Many of these studies integrated remote sensing, Geographic Information System (GIS) and the RUSLE approach for the estimation of SE^50–55^. Other recent techniques such as Artificial Neural Networks or Machine Learning techniques are also becoming popular for erosion simulation and modelling in Iran^29,56,57^. However, despite the numerous studies on SE estimation, there is limited information on SE estimation based on future climate change (CC) scenarios in Iran and other arid and semi-arid countries. Thus, the main goals of this research are to 1) estimate the current SE in the central part of Iran, and 2) predict SE changes under future climate scenarios using Climate Model Intercomparison Project-5 (CMIP5). We hypothesize that this will provide important information for policymakers and stakeholders to develop appropriate strategies to achieve sustainable land resource planning, utilization and management.

## Material and methods

### Study area

**Figure 1:**
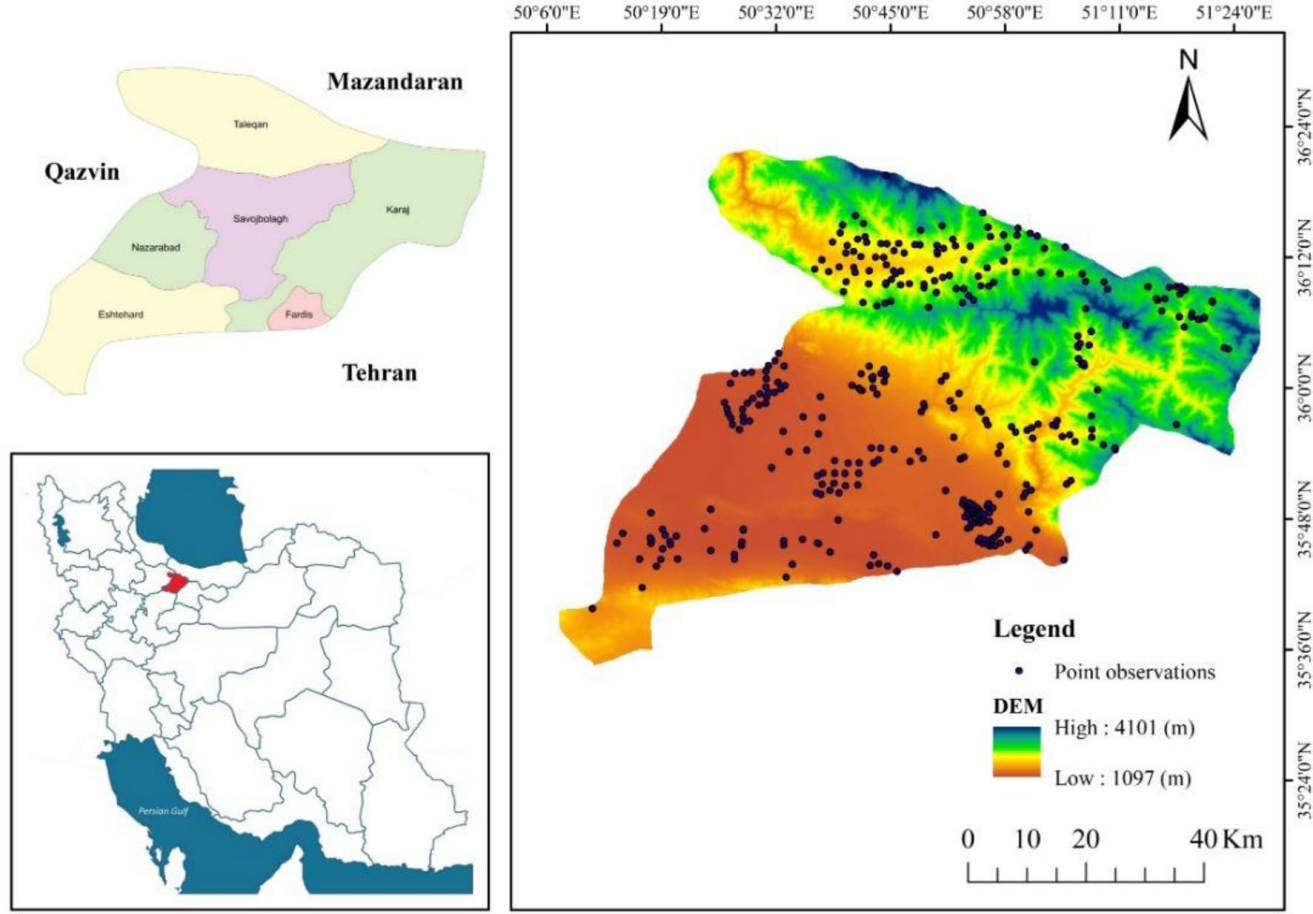
Study area with soil sampling sites shown on the Digital Elevation Model (DEM), values of pixels were mapped by ArcGIS 10.5 (https://www.esri.com/en-us/about/about-esri/overview).

This study was conducted in an area covering 5833 km^2^ in Alborz Province located in central Iran. The area lies between the latitude 35° 31′–36° 21′ N and longitude 50° 10′–51° 30′ E (Fig. 1). The climate of this area is classified as semi-arid bordering to arid^58^. Mean annual rainfall reaches 251 mm and the mean monthly temperature 14.1 °C. During the year, the temperature typically varies from − 2 to 35.2 °C and the precipitation ranges from 1 or 2 mm to 78 mm in the rainy month (https://weatherspark.com). The study area is characterized by a range of land use and land cover categories including rangeland, agricultural land, saline and bare lands.

### Data collection and pre-processing

The soil database was elaborated for several years by the Soil Science Department of the University of Tehran. To predict the spatial distribution of soil texture and soil organic carbon of the study area; Decision Tree (DT) and Artificial Neural Network (ANN) models were generated using 70% of data obtained from laboratory analysis of soil samples. Model testing was based on 15% of the data while 15% was used for model validation. As the performance of the DT is better than ANN (Appendix 1 and 2), the output of the DT model was adopted as the main input for calculating the K factor^59^.

Digital elevation models (DEM) and cloud-free Landsat images of the study area were obtained from the United State Geological Survey (USGS) website at (https://earthexplorer.usgs.gov). The images obtained include Landsat 7 Enhanced Thematic Mapper Plus (ETM +) Level-1 of 27 October 2005, 10 November 2010, 24 November 2015 and 18 October 2019. SCL-off error in the images was corrected using Landsat Toolbox extension in ArcGIS 10.5 (https://www.esri.com/en-us/about/about-esri/overview) (ESRI, USA).

Downscaled CMIP5 monthly precipitation parameter was acquired from the WorldClim Data Portal at 1 km resolution (https://www.worldclim.org/). The CMIP5 data from Global Climate Models (GCMs) are available for four representative concentration pathways (RCPs) as released by the twenty-first century in its 5th Assessment Report (IPCC, 2014). The GCM outputs have been downscaled and calibrated, (i.e. bias-corrected using WorldClim v.1.4 as current baseline climate)^60,61^. Previous research has shown that different CC scenarios produced different result in assessments of GCMs performance in Iranian territory^62,63^. In this research, the HadGEM2-ES model was used for the calculation of rainfall erosivity R-factor. For an investigation of the impacts of future CC on SE by water, the output layers of the current climate and projected CC according to the HadGEM2-ES model and several RCP (RCP2.6, 4.5, and 8.5) were used in our calculation of the *R* factor.

### Soil erosion estimation

In this study, RUSLE^31,64^ was used for estimating and predict SE. This is one of the universal pioneer methods for SE estimation and modelling^65^. It is recognized as an empirical model limited to calculating rill and inter-rill erosion, without considering gully erosion^21^. SE estimation using RUSLE is based on the following Eq. (1):

1 $$\vartheta = {\text{R}} \cdot {\text{K}} \cdot {\text{LS}} \cdot {\text{C}} \cdot {\text{P}}$$

where $\vartheta$ represents the annual soil loss (metric tons per hectare per year); *R* is the rainfall erosivity (megajoule millimeters per hectare per hour per year); *K* means soil erodibility (metric ton hours per megajoules per millimeter); *LS* corresponds to the topographic factor (length and steepness- unitless); *C* is the land cover/ land use factor (unitless); and, *P* characterizes support/conservation practice (unitless).

#### Rainfall erosivity R

*R* factor refers to the kinetic energy of raindrops which could significantly affect the stability of soil aggregates and enhance soil loss^66–70^. In this study, the *R* factor was calculated using a monthly database approach as following^31,71–74^ in Eq. (2):

2 $${\text{R}} = { }\mathop \sum \limits_{{{\text{i}} = 1}}^{12} 1.75 \times 10^{{\left( {1.5{ }\log_{10} \left( {\frac{{{\text{p}}_{{\text{i}}}^{2} }}{{\text{P}}}} \right) - 0.8188} \right)}}$$

where *R* is a rainfall erosivity factor (MJ mm ha^−1^ h^−1^ per year); $p_{i}$ represents monthly rainfall (mm); and, $P$ corresponds to the annual rainfall (mm).

The *R* factor was calculated for two different periods to account for the past, current and future values. The initial value of the *R* factor was obtained by computing the average values from 1990–2019 (R_average (1990–2019)_) and was considered as a representative average result for the past and current time interval. For future climate projection, two different average values were selected for the *R* factor. The first one was from 2040–2060 (R_average (2040–2060)_) and the second one from 2060–2080 (R_average (2060–2080)_).

#### Soil erodibility *K*

*K* factor reflects the susceptibility of soil aggregates to detachment by raindrops and its transportation by runoff^75–77^. The K values were calculated using soil data derived from the DT model simulation^78^ based on Eq. (3):

3 $${\text{K}} = 0.2 + 0.3{\text{e}}^{{0.02 \times {\text{SAN}}\left( {1 - \frac{{{\text{SIL}}}}{100}} \right)}} \times \left( {\frac{{{\text{SIL}}}}{{{\text{CLA}}}}} \right)^{0.3} \times \left( {1 - { }\frac{{0.25{\text{ OM}}}}{{{\text{OM}} + {\text{ e}}^{{\left( {3.72 - 2.95{\text{ OM}}} \right)}} }}} \right) \times \left( {1 - { }\frac{{0.7 \times {\text{SN}}_{1} }}{{{\text{SN}}_{1} + {\text{ e}}^{{\left( {22.9{\text{ SN}}_{1} - 5.51} \right)}} }}} \right)$$

where *SAN:* sand%, *SIL*: silt%, *CLA*: clay%, OM: organic matter%, *SN*_*1*_ = 1 − *SAN*/100.

Afterwards, each pixel was assigned a *K* value in the GIS environment.

#### Topographic factor *LS*

The Slope length (*L*) and steepness (*S*) play vital roles in SE and reflect the potential contribution of topography in runoff and SE^65^. The *LS* factor was computed using the following equation^79,80^ (Eq. 4):

4 $${\text{LS}} = \left( {{\text{FlAc}} \times \frac{{{\text{Cell}}\;{\text{size}}}}{22.1}} \right)^{0.4} \times \left( {{\text{sin}}\;{\text{slope}} \times 0.896} \right)^{1.3}$$

where *FlAc* is the flow accumulation (contributing to the upslope area to a given cell) with a cell size of 30 m. Flow accumulation map was derived from DEM in the ArcHydro extension of the spatial analyst tool.

#### Cover management factor (*C*)

The *C* factor plays a vital role against SE by protecting the soil surface from the direct effect of raindrops, where erosion is significantly correlated with vegetation coverage^72,81,82^. In this study, the *C* value was generated by applying Eq. 5, which is based on the Normalized Difference Vegetation Index (NDVI) as follows^83^:

5 $${\text{C}} = \exp \left( { - \forall \frac{{{\text{NDVI}}}}{{\upgamma - {\text{NDVI}}}}} \right)$$

where $\forall$ = 2 and $\gamma$ = 1. Although there are other three approaches for determining the *C* factor, the remote sensing approach based on NDVI has been widely used^13,84,85^. The average *C* factor (C_x_) of C_2005_; C_2010_; C_2015_ and C_2019_ was calculated and used as a constant input for addressing the impact of climate projection on SE. It is worth to mention here that satellite images were collected in November and April for each target year, then the average NDVI was calculated for both images to get a representative image for each target year. This approach was undertaken to overcome the fact that NDVI varies widely throughout the year because it is affected by vegetation growth dynamics.

#### Support practice factor *P*

The *P* factor refers to soil loss from up and downslope tillage under specific supporting practices. For instance, contouring agriculture, strip-cropping and terracing affect the direction of surface runoff and modify flow pattern^86,87^. In this study, the *P*-factor map was derived from DEM and the appropriate value was assigned to each category of the slope following Morgan^88^ (Table 1).

**Table 1:** The *P* factor value for different slope gradients.

Slope (%)	*P* factor
9–12	0.6
13–16	0.7
17–20	0.8
21–25	0.9
> 25	0.95

The spatial pattern of SE was derived by multiplying all the factor together (pixel-by-pixel) to generate a current and future SE map of the study area. In terms of future SE, *LS*, *K*, *P* factors were considered as constant (similar to the current situation), *C* factor was calculated as an average of (C_2005_, C_2010_, C_2015_, C_2019_), while *R* factor was estimated as an average for two different periods the 2050s and 2070s. Hence, the future erosion model could be express ass follow:

6 $${\text{RCP}}2.6,\;{\text{RCP}}4.5,\;{\text{RCP}}8.5{:}\;\upvartheta = {\text{R}}_{{{\text{average}}\;2040 - 2060}} \cdot {\text{K}} \cdot {\text{LS}} \cdot {\text{C}}_{{({\text{average}})}} \cdot {\text{P}}$$

**Figure 2:**
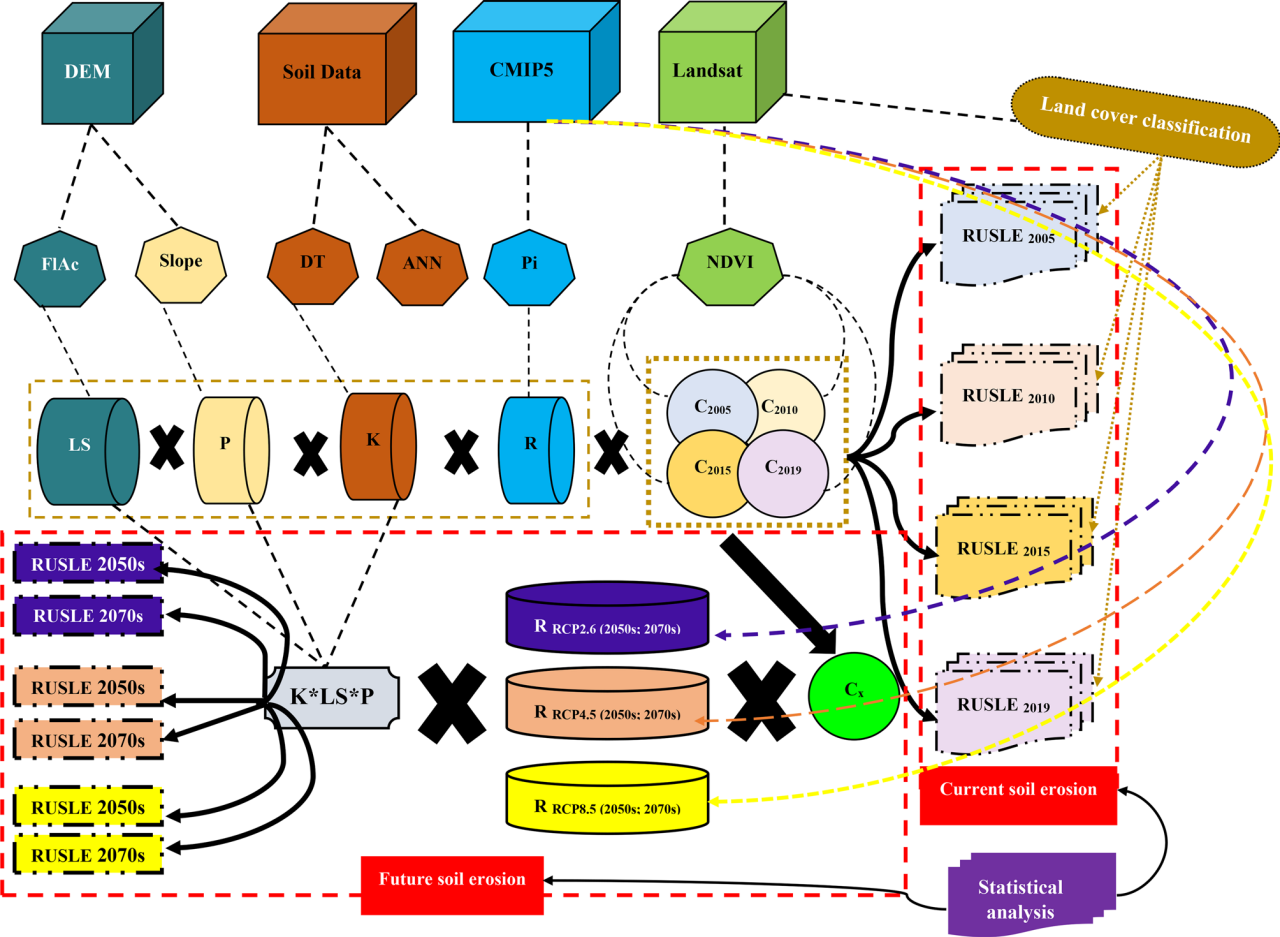
Methodological flowchart for modelling SE in the central part of Iran.

The methodology was represented in a flowchart in Fig. 2, and Table 2 shows a summary of the data sources used in this study.

**Table 2:** Data description and sources.

Factor	Type	Format	Spatial resolution	Source
*R* factor	1970–2000	TIFF	1 km^2^	WorldClim v.1.4 and v2.1
	RCP 2.6			
	RCP 4.5			
	RCP 8.5			
*K* factor	Soil samples	.xlsx converted to TIFF	–	320 Soil samples and DT modeling
*LS* factor	DEM	Raster	30 m	http://opentopo.sdsc.edu/lidar?format=sd
*C* factor	NDVI_2005–2010–2015–2019_	Raster	30 m	https://earthexplorer.usgs.gov/
*P* factor	DEM	Raster	30 m	http://opentopo.sdsc.edu/lidar?format=sd

### Statistical analysis

We estimated mean values, standard deviations and mean errors for SE factors and total erosion rates using the Extract Values by Points’ tool of ArcGIS 10.5 (https://www.esri.com/en-us/about/about-esri/overview ). Finally, the correlation between total SE and each respective factor was determined using a correlation matrix.

## Results

### Factors influencing soil erosion

**Figure 3:**
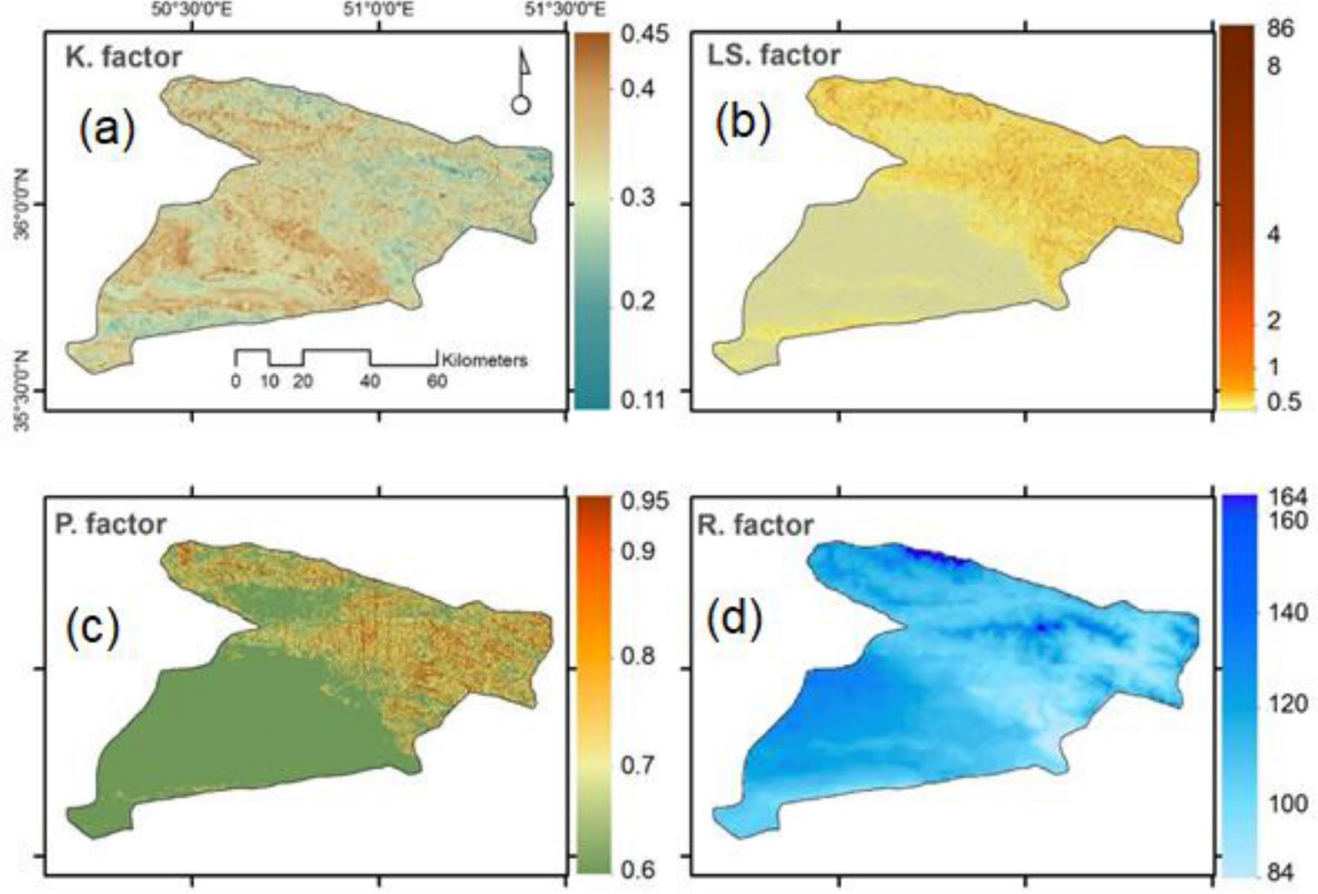
Spatial pattern of (**a**) K factor, (**b**) LS factor, (**c**) P factor, (**d**) R factors in Central Iran, values of pixels were mapped by ArcGIS 10.5 (https://www.esri.com/en-us/about/about-esri/overview).

In this study, the soil erodibility (*K*) factor widely varied. It ranges from 0.2 t·ha·h·ha^−1^·MJ^−1^·mm^−1^ to 0.4 t·ha·h·ha^−1^·MJ^−1^·mm^−1^ with a mean value of 0.25 t·ha·h·ha^−1^·MJ^−1^·mm^−1^. This variation appears to be influenced by land use and soil type. For instance, the *K* factor values range from 0.11 t·ha·h·ha^−1^·MJ^−1^·mm^−1^ (less resistant to SE) in the northern, eastern and southern parts of the study area, to 0.45 t·ha·h·ha^−1^·MJ^−1^·mm^−1^ (most resistant to SE) the central, northwest and western parts. Erodibility is particularly high in the cultivated area (0.3–0.44 t ha h ha^−1^ MJ^−1^ mm^−1^) but lower in areas with high relief (0.25–0.01 t ha h ha^−1^ MJ^−1^ mm^−1^) (Fig. 3a).

Figure 3b shows that the mean value of the slope length (*LS*) factor is 4. In the study area, the *LS* factor ranges from 0.5 to 8.6. The *LS* value is higher in the high and dissected escarpments in northern, northwest, and northeastern parts of the study area than in the south and southwest ones that are characterized by gentle slopes and low runoff potential. Although the average *P* factor value is 2.5, more than 60% of the study area registered a low *P* factor (Fig. 3c). The high values of the *P* factor coincide with the physiography and severity of slopes in the study area.

Rainfall erosivity factor ranged between 84 MJ mm ha^−1^ h^−1^ per year in lower-lying terrain areas but increased rapidly to 164 MJ mm ha^−1^ h^−1^ per year at higher terrain (Fig. 3d). The annual mean of R-value in the study area was 112 MJ mm ha^−1^ h^−1^ per year.

**Figure 4:**
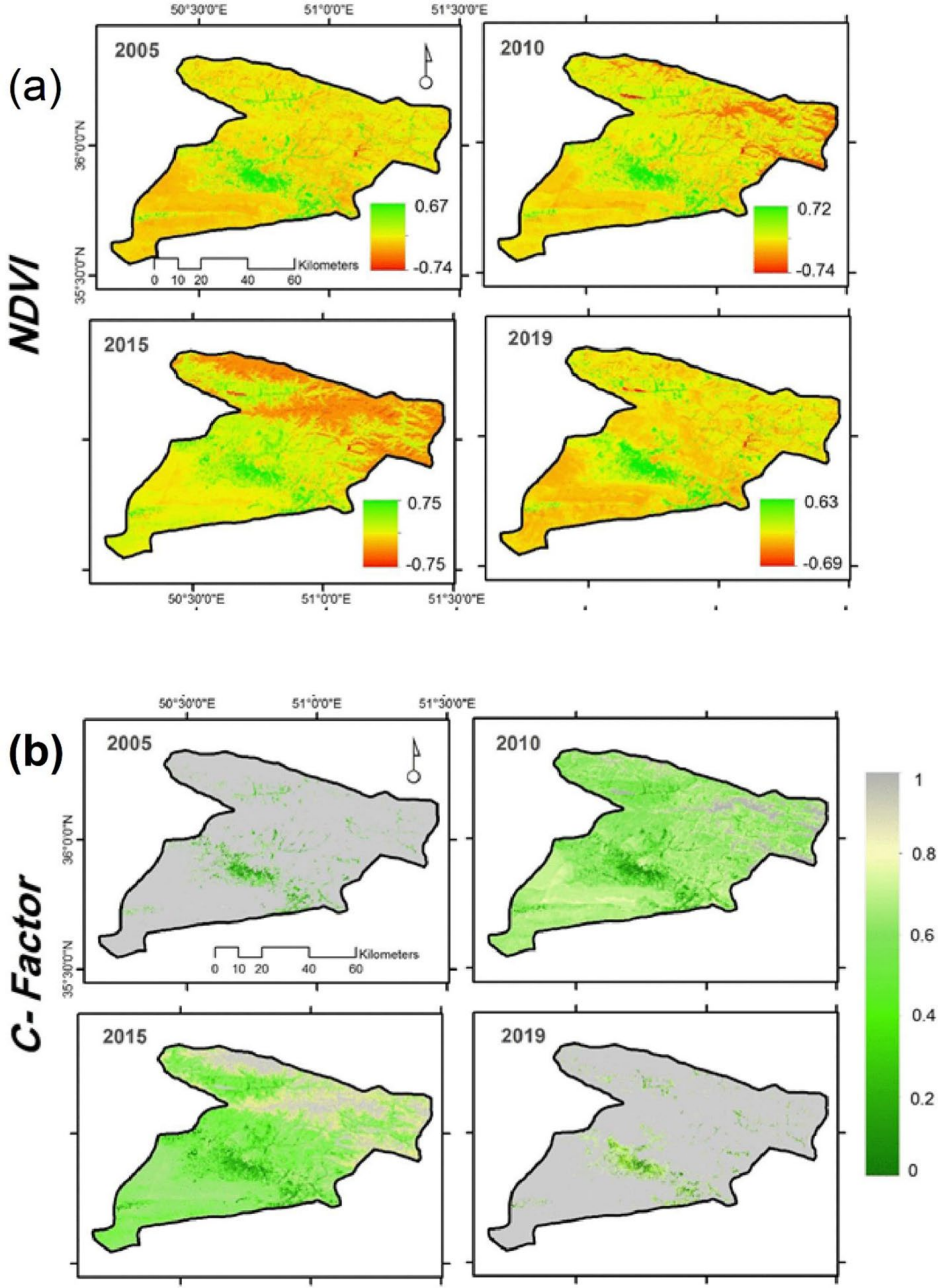
(**a**) NDVI; and (**b**) Cover management (C-factor) distribution (2005; 2010; 2015; 2019), values of pixels were mapped by ArcGIS 10.5 (https://www.esri.com/en-us/about/about-esri/overview).

Figure 4 shows the normalized difference vegetation index (NDVI) and the land cover management factor in the study area. Variation in NDVI values (Fig. 4a) during the period of this study were low in marked contrasts to the values of cover management distribution factor. Figure 4b shows *C* factor values in 2005, 2010, 2015, and 2019. There was a remarkable difference in the *C* factor during the period of this study. This is particularly significant in 2005 and 2019 and 2010 and 2015. Consequently, the land covers during 2010 and 2015 are the most vulnerable to increasing trend of erosion.

### The spatial pattern of soil erosion

**Figure 5:**
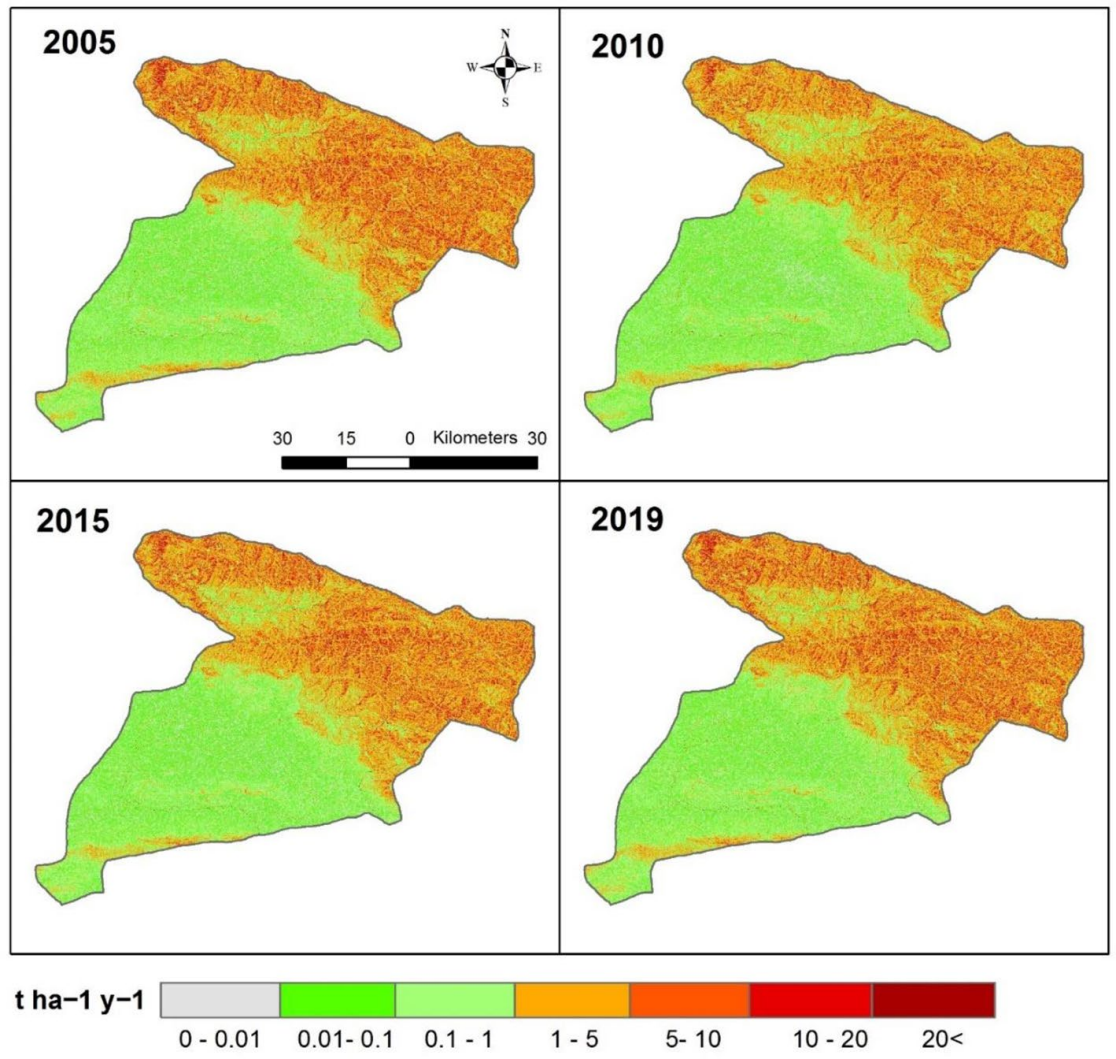
Spatial pattern of soil erosion in the study area, values of pixels were mapped by ArcGIS 10.5 (https://www.esri.com/en-us/about/about-esri/overview).

Figure 5 shows that the estimated annual rate of SE in the study area during 2005, 2010, 2015 and 2019 reaches approximately 12.8 t ha^−1^ y^−1^. The northern region of the study is more prone to soil erosion as this part of the study area accounts for more than 20 t ha^−1^ y^−1^ of erosion in contrast to the southern parts > (1 t ha^−1^ y^−1^).

Table 3 indicates the area affected per SE categories (%) under both the current and projected climate change scenarios. The annual soil loss in most parts of the region range between 0.1 and 5 t ha^−1^ y^−1^.

**Table 3:** Area affected per SE categories (%) under current climate and climate change projection.

	*ϑ* Baseline 1970–2000				*ϑ* RCP 2.6		*ϑ* RCP 4.5		*ϑ* RCP 8.5	
	2005 (%)	2010 (%)	2015 (%)	2019 (%)	2040–2060 (%)	2060–2080 (%)	2040–2060 (%)	2060–2080 (%)	2040–2060 (%)	2060–2080 (%)
**ϑ categories (t/h/y)**										
0	4.96	4.96	4.96	4.96	4.96	4.96	4.96	4.96	4.96	4.96
0.01–0	3.95	5.15	4.97	3.83	3.74	3.45	3.64	3.73	3.73	3.37
0.1–0.01	16.68	18.63	18.86	16.63	16.35	15.41	16.08	16.34	16.38	15.22
1–0.1	27.76	28.06	27.43	27.88	28.32	27.88	28.64	28.17	28.62	28.25
5–1	28.81	29.67	28.55	28.86	29.87	28.65	30.18	29.63	30.26	29.11
10–5	12.02	9.6	10.48	12.02	11.54	12.83	11.45	11.74	11.21	12.63
20–10	4.64	3.18	3.81	4.64	4.17	5.37	4.05	4.33	3.87	5.1
> 20	1.18	0.75	0.95	1.18	1.05	1.45	1	1.1	0.95	1.36

*ϑ* is annual soil loss (metric tons per hectare per year).

Table 4 shows the matrix of statistical correlation between RUSLE criteria and the values of SE in the study area. The table indicates that although slope length and management practices are correlated with SE in the study area, slope length has a greater influence on SE than management practices. This is in marked contrast with *R*, *K* and *C* factors that seem to be more fragile in relation to semi-dry physiographic features in the study area.

**Table 4:** Matrix between correlation SE variables and *ϑ* on a pixel level (n = 5,683,158).

	*ϑ*	C-Factor	K-Factor	LS-Factor	P-Factor	R-Factor
*ϑ*	1	0.057	− 0.003	**0.77**	0.31	− 0.05
*C*-Factor		1	− 0.06	0.03	0.11	− 0.02
K-Factor			1	− 0.07	− 0.21	0.07
*LS*-Factor				1	0.23	− 0.08
*P*-Factor					1	− 0.14
*R*-Factor						1

All *r* values are significant at 95% probability level, where for a sample size of the 5.7 million at 2-tailed person correlation; the r critical value is 0.0008; i.e. all the correlation values within the table are significant (p < 0.05).

### Projected soil erosion

**Figure 6:**
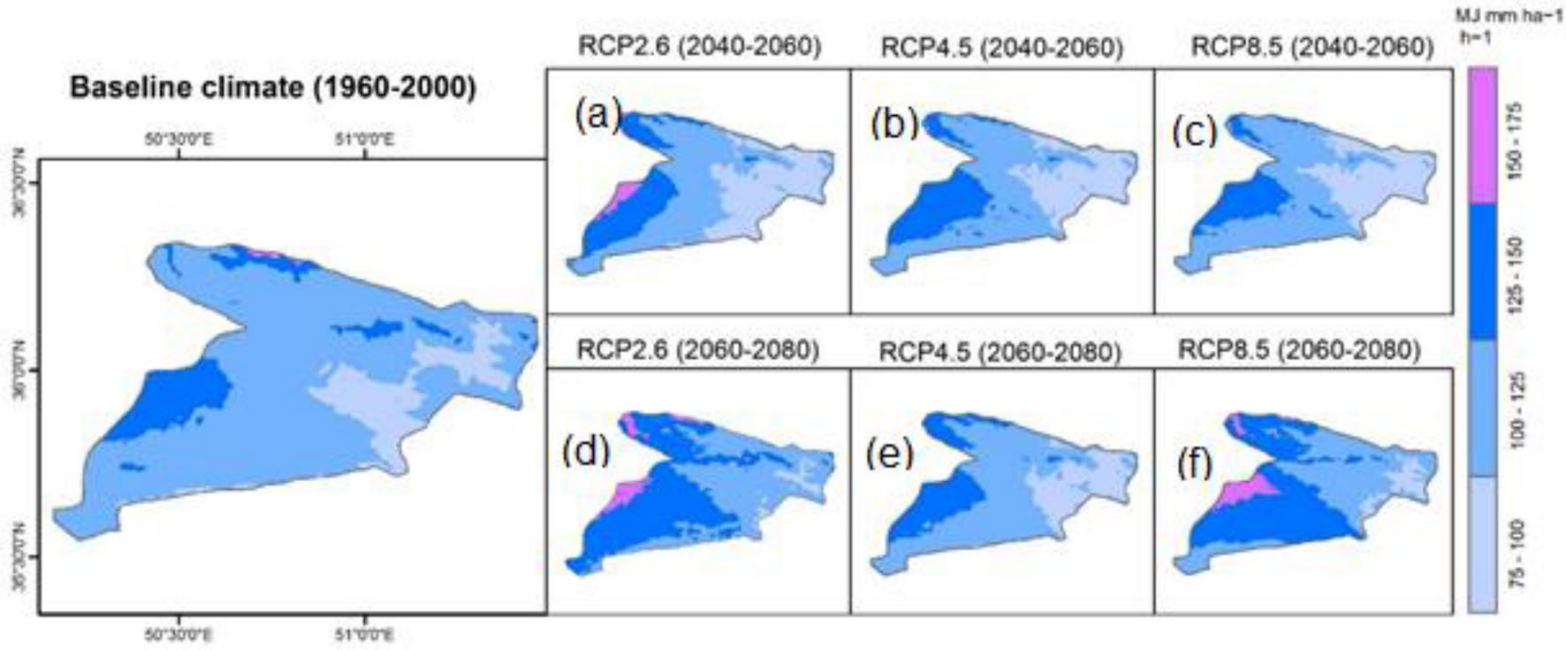
Projected changes in R factor values based on the HadGEM2-ES model and RCP for different time series, values of pixels were mapped by ArcGIS 10.5 (https://www.esri.com/en-us/about/about-esri/overview).

**Figure 7:**
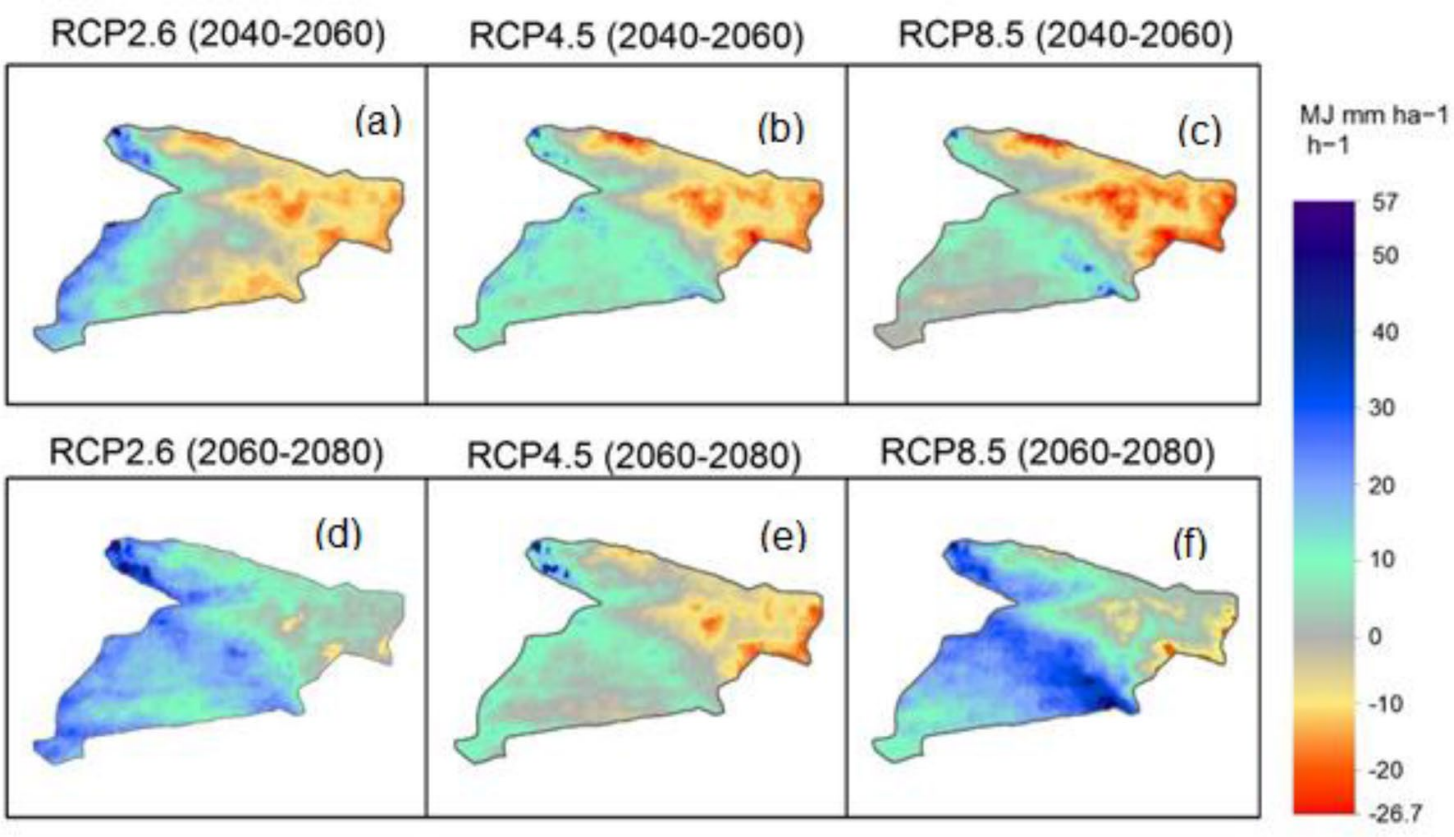
Projected future changes of R factor compared with the baseline (1970- 2000), values of pixels were mapped by ArcGIS 10.5 (https://www.esri.com/en-us/about/about-esri/overview).

Three scenarios of projected *R* factor for 2040–2060 and 2060–2080 were determined from the fifth phase of the Coupled Model Intercomparison Project (CMIP5) models. A comparison of the baseline projected *R* factor values calculated from monthly rainfall rates of 40 years (i.e. 1960–2000) with the future values of projected R factor derived from three Representative Concentration Pathways (RCPs) is shown in Fig. 6. The highest values of the *R* factor (< 150 MJ mm ha^−1^ h^−1^) are mainly concentrated in the west (a) and northwestern (b and c) part of the study area. A similar pattern is observed in the projected values (Fig. 6d–f). Figure 7 shows the changes between baseline and projected SE in the study area under three CC scenarios of RCPs. This confirms that the regions with higher values of the *R* factor are located in the eastern and northeastern regions.

**Figure 8:**
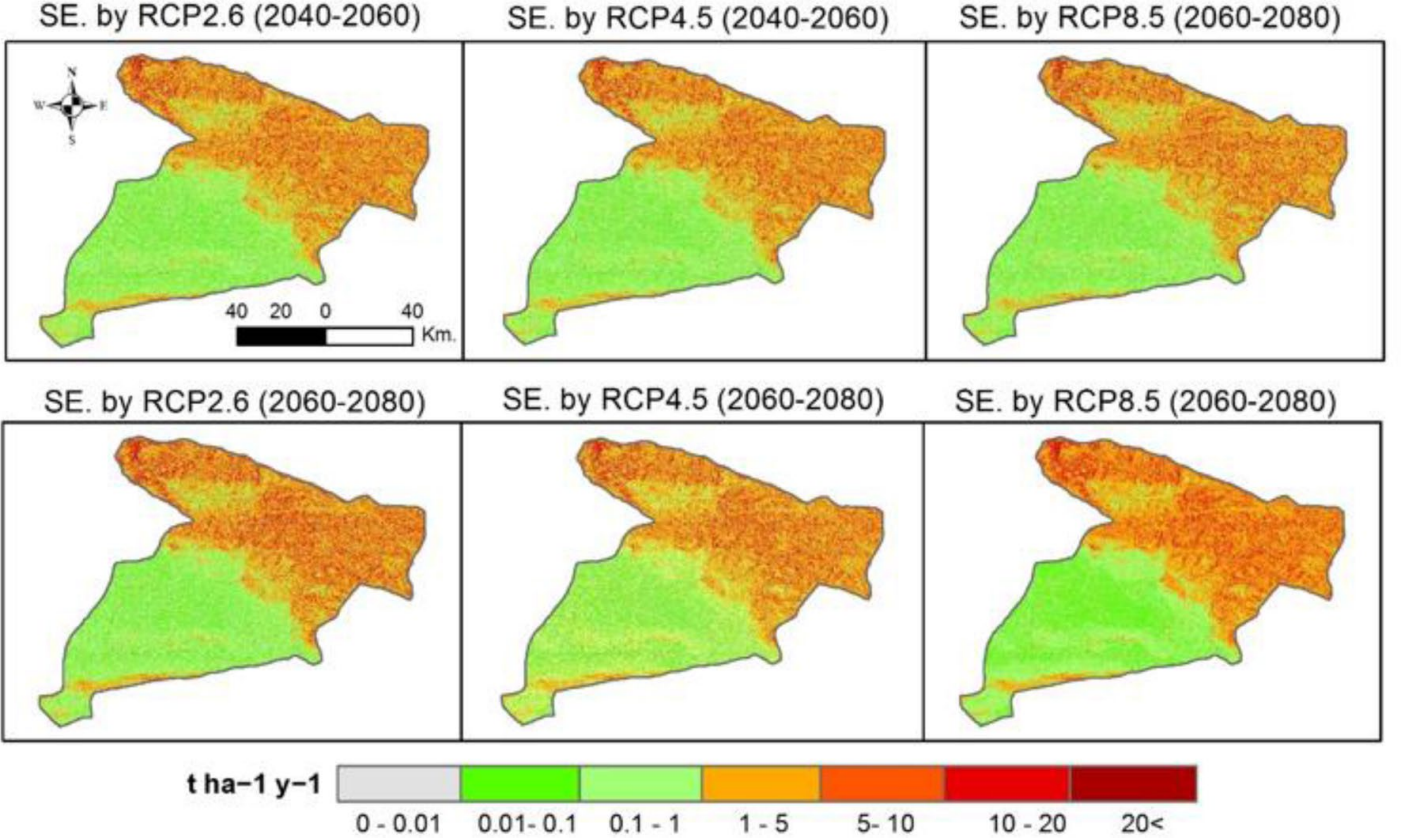
Projected future SE changes under three climate scenarios of RCPs (SE: Soil Erosion), values of pixels were mapped by ArcGIS 10.5 (https://www.esri.com/en-us/about/about-esri/overview).

**Figure 9:**
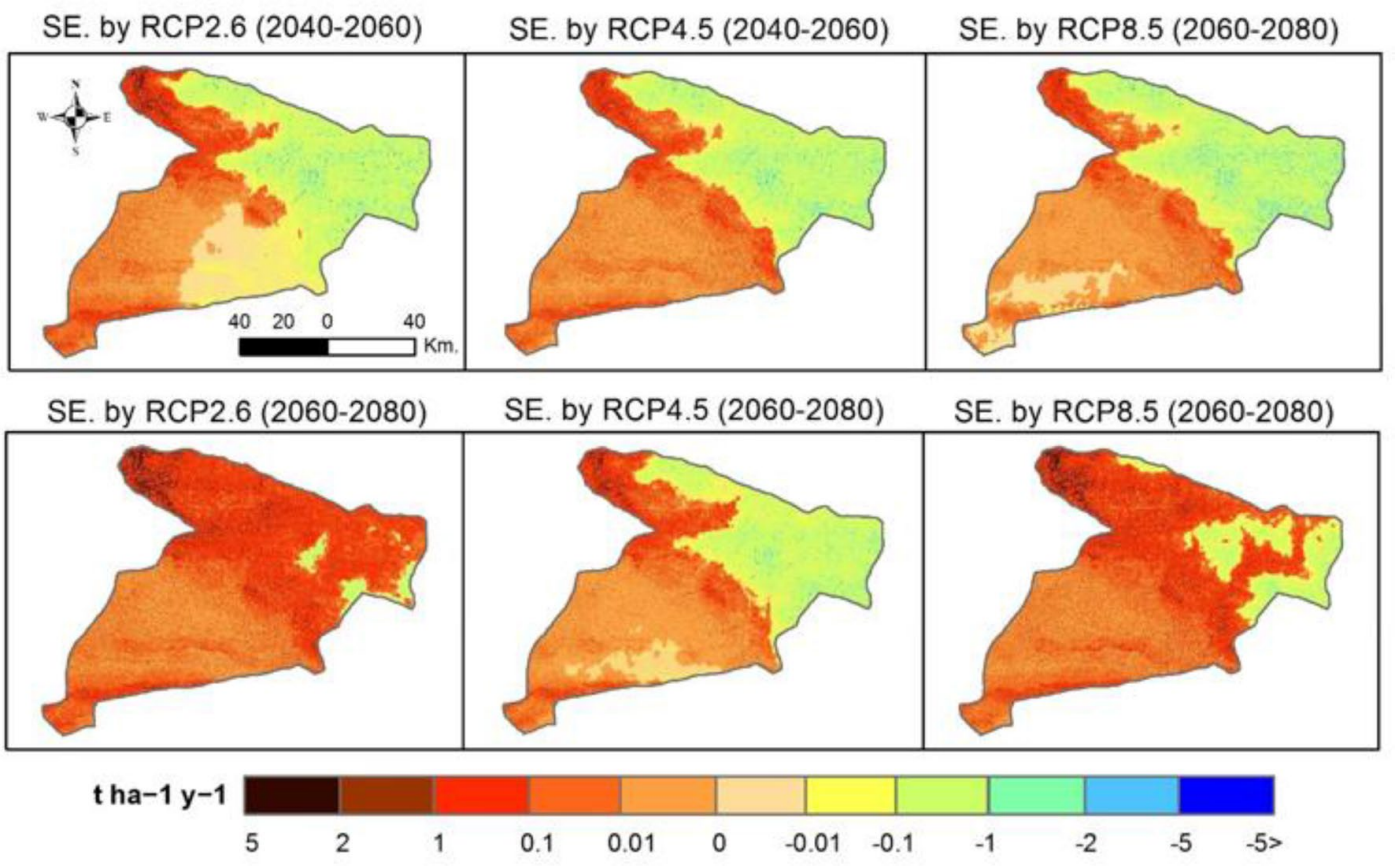
Spatial differences between RUSLE under different RCPs scenarios and RUSLE-2019, values of pixels were mapped by ArcGIS 10.5 (https://www.esri.com/en-us/about/about-esri/overview).

Projected future SE values indicate that there will be a high soil loss (> 5 t ha^−1^ y^−1^) in the north, northwest, and far southern parts of the study area in three scenarios of RCPs (Fig. 8). These future changes show that the spatial distribution of SE is similar to the baseline values (Fig. 5). This future simulation indicates that those same areas would be subject to accelerate SE if adequate soil conservation strategies are not developed and implemented. Notably, areas of high erosion values (> 5 t ha^−1^ y^−1^) reach up to 19.7% in the RCP2.6 (2060–2080) and 19.1% in the RCP8.5 (2060–2080) (Table 3 and Figs. 8 and 9). Spatial differences between RUSLE under different RCPs scenarios and RUSLE-2019 show an accelerated erosion trend in most areas of the study area. The highest values of SE were mainly located in the northwestern parts for the three RCPs (re-coloured), and especially under the RCP8.5 scenario.

### Detecting the land uses prone to SE

Table 5 shows the results of baseline and future SE quantities according to the land cover types in the study area. There is an upward trend in the quantities of SE. Rangeland areas accounted for the highest amount of SE especially in RCP2.6 (2070) and RCP8.5 (2070) with an overall amount of 4.25 t ha^−1^ y^−1^ and 4.1 t ha^−1^ y^−1^, respectively. Then, they are followed by agricultural areas with 1.31 t ha^−1^ y^−1^ (RCP2.6 in 2070) and 1.33 t ha^−1^ y^−1^ (RCP8.5 in 2070). Also, bare land areas were predicted to register up to 0.34 t ha^−1^ y^−1^ and 0.35 t ha^−1^ y^−1^ in RCP2.6 (2070) and RCP8.5 (2070), respectively. The lowest amount of SE was estimated in the residential areas reaching 0.61 t ha^−1^ y^−1^ and 0.63 t ha^−1^ y^−1^ in RCP2.6 (2070) and RCP8.5 (2070), respectively.

**Table 5:** Zonal statistical (Mean t ha^−1^ y^−1^, Std.) for each land cover under current climate and projected CC according to HadGEM2-ES model and several RCP (i.e. RCP 2.6, 4.5, and 8.5).

L.C		Rangeland		Agricultural		Residential		Bare land	
Statistic		Mean	Std	Mean	Std	Mean	Std	Mean	Std
Baseline		3.91	6.88	1.15	11.4	0.53	1.9	0.3	1.31
RCP 2.6	2050s	3.72	6.97	1.19	13.1	0.51	1.8	0.32	1.36
	2070s	4.25	7.7	1.31	13.6	0.61	2.1	0.34	1.46
RCP 4.5	2050s	3.67	6.71	1.17	12.1	0.55	1.9	0.32	1.35
	2070s	3.8	6.94	1.2	12.5	0.54	1.9	0.31	1.32
RCP 8.5	2050s	3.6	6.62	1.15	12.1	0.55	1.9	0.31	1.32
	2070s	4.1	7.6	1.33	13.8	0.63	2.2	0.35	1.5

Table 6 summarizes the changes in statistical parameters from the baseline for each land cover. SE was projected to increase in the 2070s under both RCP2.6 and RCP8.5. In contrast, predicted SE in RCP 4.5 is expected to decline in rangelands by − 0.24 t ha^−1^ y^−1^ (the 2050s), and − 0.18 t ha^−1^ y^−1^ (the 2070s) but it would slightly increase for the other land-use types.

**Table 6:** Changes of statistical parameters from the baseline.

L.C		Rangelands		Agriculture		Residential		Bare lands	
Statistic		Mean	Std	Mean	Std	Mean	Std	Mean	Std
RCP 2.6	2050s	− 0.19	0.6	+ 0.02	0.37	− 0.02	0.18	+ 0.02	0.1
	2070s	+ 0.34	0.56	+ 0.13	0.45	+ 0.07	0.24	+ 0.04	0.14
RCP 4.5	2050s	− 0.24	0.58	+ 0.017	0.28	+ 0.02	0.18	+ 0.02	0.08
	2070s	− 0.18	0.5	+ 0.031	0.3	+ 0.01	0.13	+ 0.01	0.04
RCP 8.5	2050s	− 0.31	0.63	+ 0.003	0.27	+ 0.01	0.19	+ 0.01	0.03
	2070s	+ 0.22	0.57	+ 0.14	0.47	+ 0.1	0.3	+ 0.05	0.17

## Discussion

In the semi-arid regions of Iran, SE by water is one of the most complex environmental problems threatening agricultural fields and, subsequently, human well-being. SE in these areas has been evaluated by several studies dealing with water erosion. However, there is a limited number of investigations that have rarely approached the topic of SE rates prediction according to climate covariates across remote areas^89^. In the current study, the impact of future CC on SE was investigated in the semi-arid central part of Iran featured by fragile and motivating properties for land and biodiversity degradation. We did not consider wind erosion in this study, but it is relevant to highlight that future approaches, should combine both water and wind types^90^. The five RUSLE factors (*R*, *K*, *C*, *LS*, and *P*) were extracted utilizing information from field survey and remote sensing sources. Then, these thematic raster layers were modelled and merged in the GIS environment to calculate the annual rates of SE and considers the spatial–temporal dimensions of SE in the Central Part of Iran. In this regard, given the integration of improved methods in effectively calculating erosion with recent data sources, the provided SE values by water could indicate an elevated accuracy and objectivity considering others obtained from prior studies (Table 7).

**Table 7:** Some erosion studies in different parts of Iran.

Location in Iran	Study area	Model	Type	Land cover	Rainfall (mm)	Total erosion (t/ha/yr)	References
Central	Ghareh Aghach Basin	Erosion Potential Model (EPM)	Simulated	Rangeland	358	140.69	Amiri^91^
Northern	Talar Catchment	RUSLE	Simulated	Forest and rangeland	540	92.01	Mohammadi et al.^54^
Western	Cham Gardalan watershed	RUSLE	Simulated	Rangeland and forest	592.78	38.81	Arekhi et al.^51^
Southern	Semikan watershed	RUSLE	Simulated	Rangeland, forest and arable land	308	5.7	Melo^92^
North West	Hashtrood	USLE	Erosion plots and simulation	arable land	322	1.51 t	Vaezi et al.^93^
North East	Shirindareh River Basin	IntErO and EPM	Simulation	Rangeland	318.6	2.41	Behzadfar et al.^94^
Different parts	Different	Cs-137 method	Observed	Rangeland	More than 250	30.68	Khajavi et al.^95^
Different parts	Different	Cs-137 method	Observed	Forest	More than 500	17.41	Khajavi et al.^95^
Different parts	Different	Cs-137 method	Observed	Dry open land	Less than 250	60.57	Khajavi et al.^95^
Southwestern	Mazayjan watershed	USPED	Simulation	Rangeland and cultivated areas	243	10	Zakerinejad and Maerker^96^
West-central	Fereydunshahr	Cs-137 method	Observed	Pasture land	600	46.4	Rahimi et al.^97^
West-central	Fereydunshahr	Cs-137 method	Observed	Cultivated land	600	80.4	Rahimi et al.^97^
Western	Ardal, Charmahal and Bakhtiari	Cs-137 method	Observed	Cultivated land	600	29.8	Abbaszadeh Afshar et al.^98^

The baseline and downscaled *R* factor in this study was computed and mapped based on monthly precipitation data obtained by WorldClim v.1.4 and v2.1data Portal at 1 km. resolution. Accurate mapping of baseline R factor values led to improved results in estimating SE, especially in an area with low annual precipitation rates and highly governed by climatic conditions. These results could give new insights, for example, to foresee especially the occurrence of rills and gullies among other SE processes because they are very sensitive to changes in rainfall patterns and human impacts^99,100^.

Based on the validated modelling process (DT and ANN) fed by the analysis results of 362 soil samples, the spatial distribution of the *K* factor was mapped. Moreover, *K* factor values are improved because of testing two reliable models in calculating soil properties based on data derived from remote sensing and extended field survey. In this context of statistical calibration, the DT model provided strong correlations in calculating the soil characteristic with regression of R^2^ above 70% in all measurements. However, there is still a further way to improve this model if we consider recent investigations. For example, in China, Wang et al.^101^ confirmed that based on the nonlinear best fitting techniques, *K* factor prediction by combining Geometric Mean Diameter based and soil organic matter (SOM). Another recent study conducted in Uruguay by Beretta-Blanco and Carrasco-Lettelier^102^ demonstrated that the implementation of soil taxonomy, chemical composition, and parent materials could increase the accuracy in linear estimations of this factor. These ideas agree with recent reviews published about soil mapping techniques which remark the importance of not obviating key properties since the soils are results of multiple and complex interactions^103–105^. In our study, the average K value was 0.25 t·ha·h·ha^−1^·MJ^−1^·mm^−1^, whereas the average OM was 1.9% and clay 27.43%, which contribute markedly to rising the K value in the study area. However, the K value in the study area in line with other studies in the Middle East and other semiarid regions. For instance, in southern Syria K value was ranged from 0.22 to 0.36 t ha h ha^−1^ MJ^−1^ mm^−1^^68^, in western Iran, was between 0.20 and 0.59 0.22 to 0.36 t ha h ha^−1^ MJ^−1^ mm^−1^^106^, while the average K value was 0.13 t·ha·h·ha^−1^·MJ^−1^·mm^−1^ in northern Turkey^107^.

The intense spatial–temporal variation of NDVI values has greatly affected the annual C factor values^108^. In this sense, C factor values in 2005, 2019 remarkably different from those in 2010, 2015, which could mainly attribute to megadrought events that hit the central part of Iran in that years^109^. However, C Factor, as the R factor, is largely sensitive to the areas characterized by a semi-arid environment and human impacts^110^. Consequently, these two factors led to the complicated and accelerated dimensions of SE as the current study showed. Observing our results, also non-agricultural must be considered when this factor changes such as residential areas or bare lands. Karpilo et al.^111^ stated that there is little consensus in the erosion-science community about the correct values of the *C* factor for the effects of various slope-protection materials. Therefore, we recommend that policymakers and stakeholders pay attention to that areas especially where both factors show drastically changes from nowadays to the simulated scenario to avoid irreparable loss of fertility (bare soils) or floods and extreme sediment discharges (urban areas).

*LS* and *P* factors were mapped based on the reclassification of the slope raster layer. These two were found to be the most influencing factors for erosion acceleration. These results agree with Panagos et al.^112^ who highlighted that this factor is quite obviated. They estimated that the *P* factor could reduce the risk of SE by 3%, with vegetation cover and stone walls obtaining the largest positive impact. However, these results can vary at different scales. Paying attention to research conducted at the hillslope scale, Rodrigo-Comino et al.^113^ estimated in a Mediterranean old clementine plantation for the *LS* factor using two pre-established algorithms and ISUM (Improved Stock Unearthing Method) that the micro-topographical changes can show frequent irregularities in SE results. The authors observed high differences among the areas predicted at the moment of furrow construction and the moment of data survey with soil mobilization rates of about 56.9 m^3^ (8.3 mm yr^−1^) in 19 years for 360 m^2^. Comparing *LS* and *P* factor maps with the final map of the RUSLE model explained that with rising length and percentage of the slope of the area, intensity and rate of soil erosion also is increased which is along with the result of Mohammadi and et al.^54^.

Multi-digital SE mapping enables calculation of the annual rate of SE which was reached to more than 20 t ha^−1^ y^−1^. The spatiotemporal variation of the resulting SE indicates that there is a spatial concentration of erosion in the northern, northeast, and northwestern regions. The given results indicate that the northern, northeastern and northwestern regions were the most affected in 2005, 2010, 2015, and 2019, respectively. These areas are characterized by mountainous terrain and steep slopes. Our results visibly confirmed that soil erosion could be easily affected by a different kind of land cover. The land use map (Appendix 3) showed that rangelands are dominated in the northern, northeastern and northwestern regions which has the highest soil erosion values, where *LS* factor ranges from 4–85 (Fig. 3b). On the contrary, bare land dominates in the gentle slope area (LS factor = 0.5–1), which minimize erosion processes. In this regard, Table 4 showed that the highest correlation (*r* = 0.77, *p* < 0.05) between topography (*LS*) and soil erosion, which confirm the eminent role of topography in developing the soil erosion process in the study area. In light of this rangeland and following agricultural regions showing the highest value of SE explosibility, demanded higher protection and management. Thus, that area should be considered in any future land conservation plan as a high priority considering topographical changes as key drivers of weather changes and SE intensity^114–116^. This finding completely confirmed the result of the research for Borrelli et al.^117^, in which they discussed in the high slope areas with rare vegetation the risk of SE is high. However, bare land has shown the least values of SE in the current situation, which located on a gentle slope (central part) in compression with other land use.

To assess the effect of future CC on SE susceptibility, data derived from CMIP5 by three scenarios of CMIP5-RCP were used in calculating future values of the projected *R* factor. However, the utilized approaches in the present study are consistent with those presented by Yigini and Panagos^118^ which assumed that future changes in precipitation values will inevitably lead to a change in SE rates globally. Future values of SE were predicted in the study area according to the regional CC concerning three scenarios of RCPs. These future values indicate that the northern, northwest and northeastern regions are the most sensitive and vulnerable to CC, especially under RCP8.5 which consistent with the pathway that involves huge amounts of greenhouse gas emissions^119^. High SE rates are located mainly along mountainous terrain; hence, it will be the most affected by changes in precipitation patterns for climatic characteristics in semi-arid areas^120^. Changes include an increase in extreme precipitation events across the study area, thus a greater precipitation intensity with increase SE potentials by runoff in the steep slope regions. Within this context, prediction of future SE is highly important to provide policymakers with appropriate tools for developing action plans against different possible soil erosion scenarios. Alewell et al.^121^, stressed the importance of modeling SE on a different scale for soil conservation planning and policy governance. Meanwhile, Borrelli et al.^117^ emphasized the importance of adaptation of conservation strategies based on RCP2.6 and RCP8.5 scenarios.

Future projections of CC in this study provide a spatial interpretation of the future SE in light of different scenarios. Despite the accuracy and quality of available results and the possibility of using them in the management of soil erosion, some inputs lead to uncertainty in present simulation outcomes. For example, the *R* factor values were calculated based on the monthly and yearly averages of precipitation (Eq. 3), based on 3 scenarios of RCPs, are still not certain, which could explain the low correlation between projected future *R* factor and SE in Table 4. However, the adopted calculation method is a suitable alternative in light of the scarcity of data required to calculate the values of the *R* factor according to the kinetic raindrop energy approach (basically, rainfall records at 15–30 min time interval). Besides this, there is a great difficulty in predicting future *C* factor with complete accuracy, because the C factor is complex and highly sensitive to environmental changes as was above-discussed. The current spatial outputs are of sufficient reliability for the K, LS and P factors. Consequently, this study presented serious and reliable spatial scenarios about the future of SE in the study area.

Considering all the factors, it is obvious that SE in the northern and northeastern parts which are dominated by rangeland, higher precipitation, and mountains (high relief topography) is suffering from severe erosion and also have the highest potential for SE. After the assessment of the research results and justifying them according to the geological factors in the study area, we concluded that geology also plays an important role in soil erosion activation at large scales but is mainly reflected in the form of the susceptibility of soil erosion (K-factor). Igneous formations that cover the north part of the study area are correlated with the minimum susceptibility of K-factor while basaltic and tuff formations, due to the lightweight and high porosity, can contribute more to soil formation processes and therefore to the soil erosion factor. In the foothill which sedimentation is the main characteristic, soil erosion has experienced a low rate too. Two parts of the study area revealed a high susceptibility, one is in the Taleghan area that the signs of massive erosion are obvious, and another one located at the Eshtehard with the evidence of gully erosion and dissolution erosion types.

Remarkably, this investigation verified the findings of other researchers in this part of Iran as well as many other regions of Iran^94,122^. The output of this research can be used to take measures of sustainable agriculture in an arid study area environment and to work on identifying priorities for spatial conservation. Also, SE could be mitigated by maintaining vegetation cover, using cover crops, reducing soil disturbance by tillage among other measures^123–126^.

## Conclusions

Soil erosion is one of the most pressing environmental issues in light of the accelerating impacts of global climate change. Multisource GIS provides an objective and advanced platform in soil erosion modeling with accurate and reliable results. Spatial correlation between climate change, soil erosion and land cover change using global models, such as RUSLE, can effectively assist in the spatial management process, especially in arid environments. In the present study, the spatial–temporal distribution of potential soil erosion in the central part of Iran was determined based on future climate change scenarios. The experimental RUSLE model was chosen based on the data specificity of the study area. This model provides the possibility to investigate the current and future spatial distributions of soil erosion rate relying on predictive data. The key findings are summarized, as follows:

1. The average R factor was 112 MJ mm ha^−1^ h^−1^ per year, P factor was 2.5, and the K value was 0.25 t·ha·h·ha^−1^·MJ^−1^·mm^−1^. The C factor was ranged between 0 and 1, while LS was 0.5–8.6.
2. The estimated annual rate of SE is approximately 12.8 t ha^−1^ y^−1^ in Central Iran.
3. Projected future SE values indicate that there will be a high soil loss (> 5 t ha^−1^ y^−1^) in the north, northwest, and far southern parts of the study area in three scenarios of RCPs
4. Rangeland areas registered the highest amount of SE especially in RCP2.6 (2070) and RCP8.5 (2070), followed by agricultural areas. Also, bare land areas were predicted to considerable SE rates
5. The lowest amounts of SE were estimated for the residential areas in RCP2.6 (2070) and RCP8.5 (2070).
6. SE will be increased in the 2070s under both RCP2.6 and RCP8.5. On the contrary, RCP 4.5 showed that the total SE was predicted to be decreased in rangelands and increased slightly under other land use.

The output of this research will help decision-makers and local authorities for developing a local plan for land conservation against SE by different climate change scenarios.

## Supplementary Information

Supplementary Information
