# Supplementary material for: CMIP5 climate projections and RUSLE-based soil erosion assessment in the central part of Iran
Source: Sci Rep. 2021 Mar 31;11:7273. doi: 10.1038/s41598-021-86618-z (PMC8012627; doi:10.1038/s41598-021-86618-z)

***Appendix 1, 2 , 3***

**CMIP5 Climate Projections and RUSLE-Based Soil Erosion Assessment in The Central Part of Iran**

Fatemeh Hateffard ^1^, Safwan Mohammed^2*^, Karam Alsafadi^3^, Glory O. Enaruvbe^4^, Ahmad Heidari^5^, Hazem Ghassan Abdo^6,7,8^, Jesús Rodrigo-Comino^9.10^

1. Department of Landscape Protection and Environmental Geography, Faculty of Science and Technology, University of Debrecen, Debrecen, Hungary.
2. Institute of Land Use, Technology and Regional Development, University of Debrecen, Debrecen, 4032, Hungary;
3. Department of Geography and GIS, Faculty of Arts, Alexandria University, Alexandria 25435, Egypt
4. African Regional Institute for Geospatial Information Science and Technology, Obafemi Awolowo University, Ile-Ife, Nigeria
5. Soil Science Department, University of Tehran, Karaj, Iran
6. Geography Department, University of Damascus, Damascus, Syria
7. Geography Department, University of Tartous, Tartous, Syria
8. Geography Department, University of Tishreen, Lattakia, Syria
9. Physical Geography, Trier University, 54286 Trier, Germany
10. Soil Erosion and Degradation Research Group, Department of Geography, University of Valencia, 46010 Valencia, Spain

Corresponding authors: ^*^ Safwan Mohammed ([safwan@agr.unideb.hu)](mailto:email@address.edu))

**Appendix 1. Measured and predicted soil data based on Decision Tree (DT) model for each soil properties (a) clay, (b) Silt, (c) sand, (d) OC**

**Appendix 2. Measured and predicted soil data based on Artificial Neural Network (ANN) technique for each soil properties (a) clay, (b) silt, (c) sand, (d) OC.**

**Appendix 3. land use in the study area**


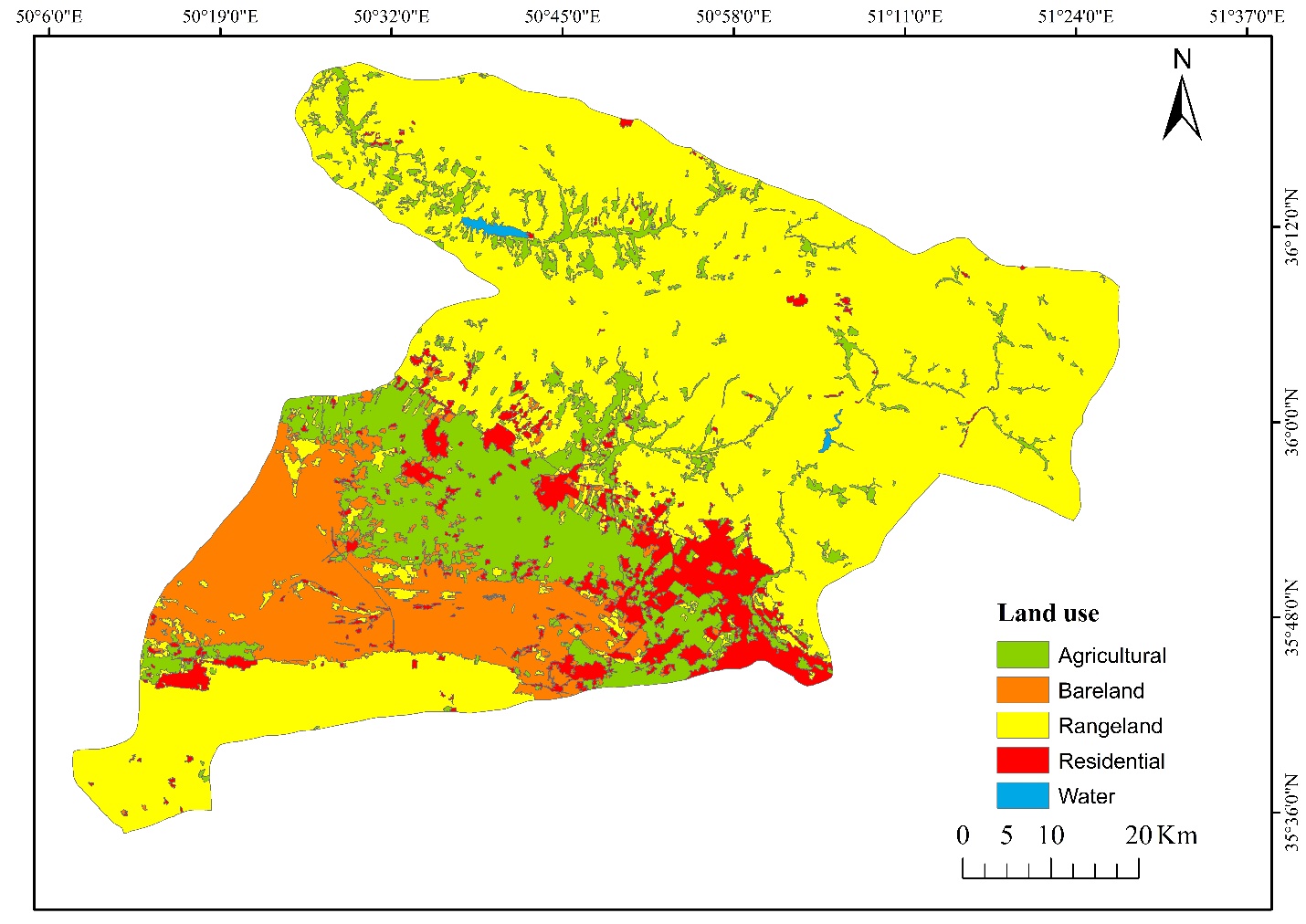

Supplement: Supplementary file 1 — Supplementary Information [file 41598_2021_86618_MOESM1_ESM.docx]
